# Stimulus Center Bias Persists Irrespective of Its Position on the Display

**DOI:** 10.3390/jemr18060077

**Published:** 2025-12-16

**Authors:** Rotem Mairon, Ohad Ben-Shahar

**Affiliations:** 1 Department of Computer Science, Ben-Gurion University of the Negev, Be’er Sheva 84105, Israel; rotem.mairon@gmail.com; 2 School of Brain Sciences and Cognition, Ben-Gurion University of the Negev, Be’er Sheva 84105, Israel

**Keywords:** eye movements, center bias, computational saliency

## Abstract

Since the earliest studies on human eye movements, it has been repeatedly demonstrated that observers fixate the center of visual stimuli more than their periphery, regardless of visual content. Subsequent research suggested only little effect of typical biases in experimental setups, such as observer’s position relative to the screen or the relative location of the cue marker. While comparative studies of the screen center vs. stimulus center revealed that both conspire in the process, much of the prior art is still confounded by experimental details that leave the origins of the center-bias debatable. We thus propose methodological novelties to rigorously test the effect of the stimulus center, isolated from other factors. In particular, eye movements were tracked in a free-viewing experiment in which stimuli were presented at a wide range of horizontal displacements from a counterbalanced cue marker in a wide visual field. Stimuli spanned diverse natural scene images to allow inherent biases to surface in the pooled data. Various analyses of the first few fixations show a robust bias toward the center of the stimulus, independent of its position on the display, but affected by its distance to the cue marker. Center bias is thus a tangible phenomenon related to the stimulus.

## 1. Introduction

An abundance of eye-tracking studies show that human eye movement behavior is subject to certain tendencies regardless of visual content. For example, it has been shown that horizontal saccades are more dominant during free-viewing conditions [1,2,3], and that humans tend to select nearby locations more often than distant ones [4,5,6].

**Figure 1:**
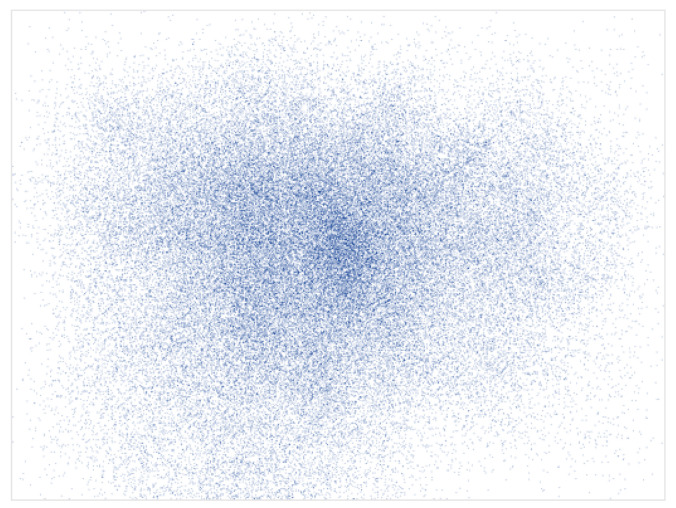
A visual representation of the center bias in the CAT2000 dataset [11], which includes 2000 images from 20 distinct categories and eye tracking data from 18 observers. All fixations from all observers recorded for all images are compiled into a single map depicting, via the spatially varying density, the tendency of observers to fixate more toward the center than the periphery.Blue dots represent individual fixations.

Perhaps the most established phenomenon in eye-tracking studies is the center bias. The term “center bias” describes the tendency of observers to fixate earlier and more frequently near the center of a stimulus presented on a computer screen rather than in its periphery (see Figure 1). This bias in the spatial distribution of human fixations was already reported in the earliest research on human eye movement behavior ([7], Chapter 1), it has been documented in a variety of scene-viewing experiments [8,9,10], and it is strongly reflected in human fixation maps [10]. In fact, its robustness across observers and images is so pronounced that many computational saliency and scanpath models formalize this bias as a simple center prior.

One immediate explanation for the center bias may be the spatial distribution of the visual content presented in stimuli. Indeed, most natural images present objects of interest more near the center of scenes than around the periphery [10], possibly because photographers tend to frame their subjects closer to the center of a scene ([12], Chapter 2). Therefore, if human fixations correlate with visual features, then a bottom-up attraction toward centrally located content would naturally emerge. At the same time, a top-down tendency for observers to fixate the centers of objects, a phenomenon known as the Preferred Viewing Location effect, Refs. [13,14,15] could also contribute to the center bias. If objects themselves are commonly centered in scenes, fixating the overall stimulus center becomes an efficient strategy for accessing object centers. That being said, the center bias has been shown to persist regardless of whether visual features are distributed at the center or the periphery [16], suggesting that centered composition of natural images cannot exclusively account for this behavioral phenomenon.

Other attempted explanations for the center bias concern the behavioral preferences of human observers. For example, observers may prefer to look “straight-ahead”, and often this direction is aligned with the center of a screen when presenting a centrally placed stimulus. Naturally, such a head direction preference may thus cause a bias toward the stimulus center. Consistent with this view, even with head-free viewing conditions, central fixation tendencies remain, though their strength can vary with task demands from the third fixation onward [17]. While this indeed reflects the conditions in most experiments, Vitu et al. [18] showed in a reading experiment that the center bias endures even when the monitor is shifted horizontally relative to the observer. This suggests that the center bias reflects a bias toward the center of the display rather than a particular head direction or related behavioral preference.

In the experiment by Vitu et al. [18], the head position was always aligned with the body position, thus limiting insights about possible effects of a relative tilt between them. Indeed, by examining the distribution of fixations when the observer’s head was either aligned or tilted horizontally relative to their body, fixations were shown to be highly correlated with the head direction [19]. Relatedly, recent real-world work shows that oculomotor and head-movement costs also influence fixation selection, with observers tending to choose movements that minimize motor effort [20]. In other words, fixations may, after all, be biased toward the location where the head is directed. Nevertheless, even when head movements are completely unconstrained, central fixation tendencies remain robust [21], suggesting that the bias cannot be attributed merely to head-movement restrictions in laboratory setups. A similar central tendency has also been observed in naturalistic, real-world viewing, where freely moving observers maintained central fixations even while actively navigating through their environment [22].

All of the above immediately raises the concern that the very settings of viewing experiments facilitate, and perhaps even cause the center bias. This is particularly reasonable considering the very similar settings used in most viewing experiments, where typically, observers sit directly in front of a computer screen that presents stimuli of natural images in the center of the display.

In addition to the above confounds, a common practice in free-viewing experiments involves using a central marker to cue observers where to fixate before stimulus onset, with the rationale of standardizing the initial viewing conditions across observers. The pre-stimulus cue directs the observer’s gaze to the cue marker before triggering stimulus onset, thus putting all observers on “equal footing” for the sake of interpreting the data. While Tatler [16] demonstrated that the cue marker alone cannot account for the center bias, Rothkegel et al. [23] showed that the timing between cue fixation and image onset plays an important role in modulating its strength.

In practice, if a saccade does not occur precisely at stimulus onset, the fixation at the cue marker’s location usually continues after the stimulus appears. This brief delay, the first saccade latency, marks the beginning of a critical temporal window during which the center bias emerges and typically reaches its peak in the earliest fixations, whereas later fixations become progressively influenced by scene content and task context [16,23]. This temporal evolution underlies our interest in the immediate post-onset fixation that we next term the *zeroth* (0th) fixation, and motivates treating the first three fixations as a window onto the dynamics of bias formation.

As will be detailed later, to account for this transition, we adopt a dedicated numbering scheme and refer to this post-stimulus-onset continuation of the cue fixation as the 0th fixation, a term we introduce in this paper, which continues until the first saccade. Unlike a conventional fixation, which begins after a preceding saccade, the unusual label “0th fixation” does not mark the start of a new fixation but rather the continuation of the one that began before the onset of the stimulus. We stress that this eye movement behavior also presents itself in all prior art studies, but the novel terminology we use here is aimed at emphasizing the organization of the gaze data from stimulus onset and its contribution to the insights we obtain later.

Indeed, to avoid incorporating trivial information into the analysis, researchers often remove this 0th fixation from the dataset and analysis. But although discarding this 0th fixation is a reasonable heuristic, the effectiveness of this strategy has never been tested rigorously, and in itself, it is unlikely to address properly the behavioral effect of a cue that is aligned with the explored bias (namely, in the center of the stimulus). We argue that by retaining the 0th fixation in our analysis, it may be possible to differentiate more easily between preparatory and stimulus-driven fixations, and labeling it this way (i.e., fixation number ‘zero’) is meant to signal its unique status.

Summarizing the above, several factors are at work in the typical experimental setting, and each of them may manifest itself in the center bias: (1) the pre-stimulus cue marker, (2) the stimulus center, (3) the display center, and (4) the observer’s head direction. Because in most prior art all these factors are typically co-centered in the observer’s field of view, determining the individual contribution of each of them to the center bias is difficult.

**Figure 2:**
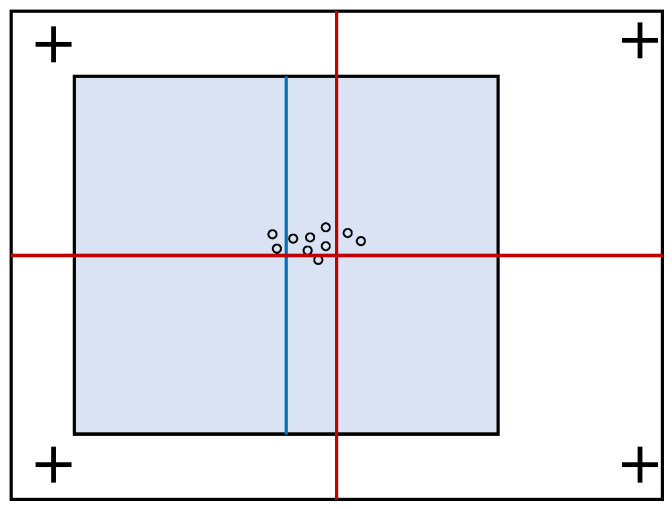
An illustration of an experimental result by Bindemann [24]. More fixations are found to the left of the screen center (red cross) and to the right of the stimulus center (blue line) when the latter is shifted to the left. Because the magnitude of the saccade was insufficient to reach the center of the stimulus, a parallel effect was concluded in which initial fixation positions are determined not only by the location of a stimulus but also by the center of the screen.

Bindemann [24] addressed this issue in an experiment to find out whether the eye is drawn to the center of the displayed scene or the center of the screen. In that experiment, stimuli with a visual angle (VA) of ∼22° × 16° were either presented at the center of the display or shifted by ∼3.7° to the left- or right-hand side. To avoid biasing the viewers to the same position at the start of each trial (as would be the case with a single central cue marker), the cue marker appeared at random in one of four positions near the corners of the display (see Figure 2).

Analysis of scan paths under these conditions showed that the first significant post-stimulus fixation, referred to in our present study as the first (1st) fixation, was drawn in the direction of the displaced stimuli. However, the magnitude of saccades was insufficient to reach the center of these stimuli. This effect was interpreted to suggest that initial fixation positions are determined by both the center of the stimulus and the center of the display. Further analyzing the effect of the cue markers on these tendencies showed that more fixations occur near the center of the stimulus when it is closest to the cue marker. However, when the stimulus center is farthest from the cue marker, observers look closer to the display center.

**Figure 3:**
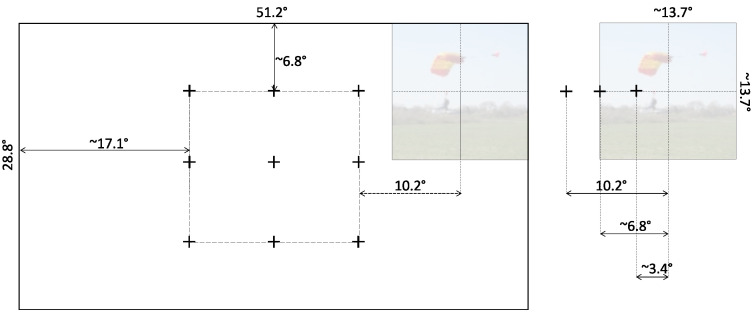
A true proportions representation in pixels of the possible locations of the stimulus on the screen display. (**Left**) Prior to the onset of the stimulus, the cue marker was displayed at one of the 9-point grid locations. The shortest distance between a marker and a screen edge allowed the stimulus to appear fully to either side of the cue marker. (**Right**) The stimulus had a resolution of 1024 pixels, which corresponded to 13.7° of viewing angle, and was displayed to the side of the marker at a distance of 256, 512, or 768 pixels (equivalent to 3.4°, 6.8°, or 10.2°, respectively).

An important aspect of the experiment by Bindemann [24] is the fact that the onscreen area of the stimuli always included the center of the display. While this could be a necessity due to physical limitations of the display and the need for stimuli of some minimal size, the relative proximity of the different factors (i.e., the center of the stimuli, center of the display, and location of the cue marker) possibly confounds the ability to separate their effects. To that end, the present study introduces a novel methodological design in which the stimulus is completely decoupled from the center of the screen. This is accomplished by employing a much higher resolution and physically larger display, reducing the size of the stimuli presented, and increasing the offset that the stimulus could take relative to the center of the screen and the cue marker position (see Figure 3). Importantly, while Bindemann [24] aimed to compare the relative influence of the stimulus and display centers, our study is not comparative in nature. Rather, we focus on testing the robustness of the stimulus center bias independently of the display center, without seeking to evaluate their relative contributions. In that sense, the ‘center bias’ in our study is thus considered the tendency of observers to fixate earlier and more frequently near the center of a stimulus, regardless of its position or location on the display. It is our goal to study and explore this definition in a rigorous methodological fashion. Finally, to further characterize the earliest steps of gaze behavior, we also examine how fixation durations relate to subsequent fixation locations, providing a temporal perspective on the transition from preparatory to stimulus-driven fixations.

## 2. Methods

### 2.1. Participants

Twenty students between the ages of 20 and 30 (mean=24.71,SD=1.55) with normal or corrected-to-normal vision took part in the experiment. All participants reported no history of neurological or ophthalmological disorders, were unaware of the purpose of the experiment, and received a small monetary compensation for their participation. All participants who started the experiment completed it and were included in the analysis. The study was reviewed and approved by BGU’s Human Subjects Research Committee and informed consent was obtained from all participants.

### 2.2. Stimuli

The stimuli were 360 RGB images chosen at random from the publicly available dataset by Judd et al. [25], which includes 1003 images of natural indoor and outdoor scenes. Stimuli thus consisted of content-rich scenes that typically include multiple objects of interest. Either the width or the height of each image was 1024 pixels, while the other dimension could vary. The selected images were cropped and/or resized to 1024×1024 pixels (while preserving the aspect ratio of visual objects in the image). Considering the viewing distance of 67 cm from the display, each stimulus image thus subtended a visual angle of 13.7° in both width and height.

### 2.3. Design

We defined a 3×3 grid of cue locations spanning the central region of the display to manipulate the spatial relationship between the cue and the stimulus (see Figure 3). Prior to the onset of each stimulus, a marker was displayed at random in one of these nine locations to cue the observers as to where to fixate. This random (but counterbalanced) location ensured that observers were not inclined to a particular screen location at the onset of each stimulus.

Once the observer fixated on the cue marker, the stimulus itself appeared (again, counterbalanced) either to the left or to the right of the marker, presented at one of three distances. Labeled *near*, *far*, and *farther*, these distances were 3.4°, 6.8°, and 10.2° of visual angle from the cue marker to the center of the stimulus, respectively.

The locations of the cue marker, as well as the appearance of the stimulus to the right or left of the marker, were pre-chosen at random to obtain the same experimental sequence for all subjects. We reiterate that regardless of the cue location, there was always enough horizontal space to position the stimulus either to its left or its right, in equal probabilities at all three distances, so the location of the marker could never prime either of these stimulus locations. In all analyses, gaze positions were expressed in degrees of visual angle relative to the center of the stimulus, based on the 67 cm viewing distance and the physical size of the display.

### 2.4. Apparatus

We used a desktop-mounted EyeLink 1000 Plus video-based infrared eye tracker (SR Research Ltd., Ottawa, ON, Canada) to record the eye movements of observers. Observers freely viewed images displayed on a 27-inch screen with a resolution of 3840×2160 (LG 27UK650-W 4K UHD UltraFine IPS Monitor with 5 ms response time and 60 Hz refresh rate), while monocular gaze position (from the better-calibrated eye, left or right) was recorded at 500 Hz. The experiment was programmed using the Experiment Builder environment (SR Research).

Segmentation of the raw gaze sequences into saccades and fixations was performed by the standard EyeLink 1000 Plus online event parser using the manufacturer’s recommended default settings. Specifically, we employed a velocity threshold of 30 deg/s and an acceleration threshold of 8000 deg/s^2^. Importantly, we did not impose an additional amplitude threshold beyond these criteria. This decision was made to capture the full continuum of eye movements, from subtle fixational eye movements to larger exploratory saccades, and to more accurately represent the natural variability in oculomotor behavior. Any eye movements not meeting the velocity and acceleration criteria were classified within a fixation, and otherwise were associated with a saccade. Notably, employing these default settings aligns with common practice in a substantial portion of the literature, especially in cognitive research contexts, where such defaults are either explicitly reported or otherwise used without being specified. See also the Section 4 for additional deliberation regarding the parsing procedure.

### 2.5. Procedure

The experiment was conducted in a dimly lit room. Participants sat in an adjustable chair 67 cm away from the display screen, with a chin rest restricting their head movements to maximize the accuracy of tracking. The experiment was divided into three sessions, each of which included 120 trials. A standard nine-point EyeLink calibration and validation procedure was performed at the start of each session, with calibration points spanning the entire display, and was repeated as needed until the average validation error was below 1° of visual angle on average.

**Figure 4:**
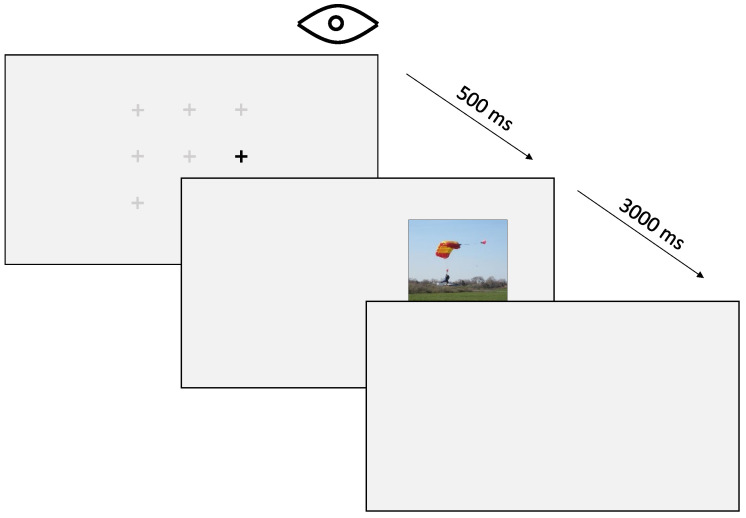
The procedure of a single trial. The observer must fixate on a pre-selected random fixation marker for 500 milliseconds before a stimulus appears on the screen. The stimulus is then displayed for 3 s before the next trial begins.

Figure 4 depicts a single trial. Each trial began with a marker to indicate where the observers should focus their initial gaze. Once the eye-tracker recorded a stable cued fixation for 500 ms, the marker itself disappeared while a scene image was displayed to the left or right of the marker location. As mentioned above, these images could appear at one of the three distances mentioned above and were displayed for 3 s while the eye tracker logged raw eye movement data.

To ensure the accuracy of our eye-tracking data, we implemented the eye-tracker’s automatic drift correction procedure. If a subject failed to maintain a stable 500 ms fixation on the cue marker for 5 s, a drift correction screen was displayed and drift correction was conducted using a single-point calibration method. Importantly, after drift correction a cue marker was presented again at its designated location and stable fixation was attempted once more before stimulus presentation. Therefore, drift correction could not directly influence fixation behavior during the period when the stimulus appeared on the screen, which is the only time when fixations were analyzed.

The average duration of trials thus varied, primarily depending on the necessity for drift correction. Typically, trials without drift correction lasted around 4 s, comprising approximately 1 s (on average) for establishing stable fixation on the cue marker and 3 s for stimulus presentation. In cases where drift correction was required, the average trial duration extended, reflecting the additional time needed for re-calibration. Across all trials, the average duration of a trial was approximately 4.38 s.

Observers were asked to look at the images presented without further instruction except for a short memory task. Specifically, to encourage observers to pay attention to the images, we stated that after the last session, a visual memory test with respect to the displayed images would be performed. In this test, observers were shown six random images and asked to identify whether or not they had seen each image during the experiment. No accuracy performance was recorded for that bogus task.

## 3. Results

To investigate the eye movement behavior following the disappearance of the cue marker and the presentation of the stimulus, we considered the 0th, 1st, and 2nd fixations separately. As already mentioned above, fixations are numbered starting from 0 to reflect the initial post-stimulus-onset continuation of the cue marker fixation (the 0th fixation), followed by subsequent fixations that actively engage with the stimulus (the 1st and 2nd fixations). Notably, this scheme also allows us to align with the prior art, as our 1st fixation corresponds to what Bindemann [24] identifies as the first fixation after stimulus onset. At the same time, this allows us to offer both preparatory data (the 0th fixation) and subsequent fixation data analysis, providing a broader temporal context.

In all analyses, trials were included for a given fixation number only if the participant produced at least that many fixations during the 3-s trial period. Under this criterion, virtually all trials contributed to the 0th fixation analysis (all but a single trial), approximately 1–2.5% of trials were excluded from the first fixation analysis, and approximately 3–5% of trials were excluded from the 2nd fixation analyses. All reported analyses are therefore based on the subset of trials that met this minimum-fixation requirement for the corresponding fixation number.

Each numbered group of fixations was further divided according to the stimulus-marker condition—either ‘near’, ‘far’, or ‘farther’. For each combination of ordination and distance, we aggregated all the fixations into a single fixation map by considering their locations relative to the stimulus in each trial. This approach of pooling fixation data is similar to that used by Bindemann [24], who also aggregated fixations to generate heatmaps fitted with a Gaussian distribution and normalized for visual comparison. In contrast, our data handling employs scatter plots that present an unmodified representation of all fixation data, allowing for a direct observation of fixation patterns without the influence of model-based smoothing.

**Figure 5:**
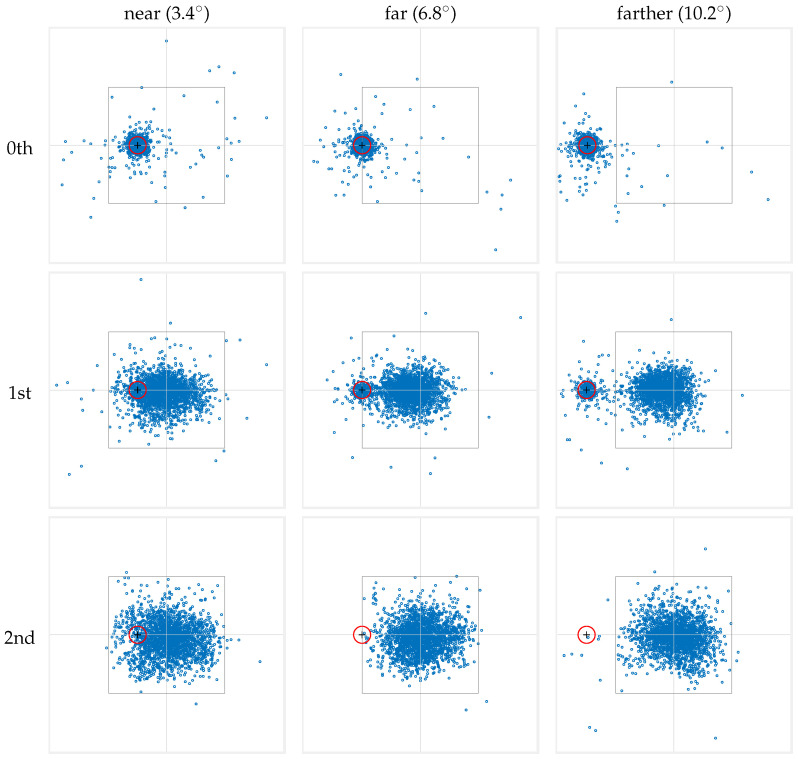
The fixations from all subjects were aggregated into distinct fixation maps based on their ordinal fixation number (rows) and stimulus-to-marker distance (columns). Fixations are numbered starting from 0, where the ‘0th’ fixation corresponds to the gaze at the cue marker. To facilitate visualization, we calculated fixation locations relative to the center of the stimulus. In each panel, the inner square denotes the on-screen area in which the stimulus was presented, with the intersection of the horizontal and vertical lines indicating the stimulus center. The red circle represents the 1-degree visual angle area around the cue marker, within which a fixation triggers the stimulus onset. The data presented consolidate both conditions where the cue marker is located to the left and to the right of the stimulus center. To achieve this, data from right-side cue marker conditions have been horizontally mirrored and combined with the left-side data.

The resulting fixation maps are presented in Figure 5, where the rows correspond to fixation numbers, and the columns represent the different marker-to-stimulus distances. The data presented consolidate both conditions where the cue marker is located to the left and to the right of the stimulus center. To this end, data from right-side cue marker conditions have been horizontally mirrored and combined with the left-side data.

### 3.1. Behavior of 0th Fixation

As mentioned, each trial begins with the observer fixating at a cue marker for 500 ms, a requirement to trigger the stimulus onset. Once this happens, the cue marker vanishes, the stimulus is presented, and gaze sequence-logging begins, all at once. However, unless the observer initiates a saccade exactly at stimulus onset, the first recorded fixation is likely a continuation of the pre-stimulus fixation at the cue marker. We refer to this post-stimulus continuation as the 0th fixation, though it differs from a classical fixation, which begins at the end of a saccade. Instead, the 0th fixation represents the lingering portion of the fixation that started before the stimulus onset but occurs after stimulation transition. Indeed, if the observer maintains fixation on the cue marker, the eye-tracker registers it as a continued fixation at that position. This pattern arises from two factors: the gaze stability (or lack thereof) just before stimulus presentation and the time required for the visual system to process the fresh stimulus for gaze programming.

As the maps in the top row of Figure 5 show, the 0th fixation is densely concentrated around the location of the cue marker. This strongly supports the common practice of discarding the 0th fixation in scanpaths that originates from a cue marker in order to avoid trivial information in fixation analysis (see the Section 1). Indeed, the observed pattern for the 0th fixation directly results from our trial procedure and the observers’ behavioral response. However, while the majority of 0th fixations are within 1° VA from the cue marker (84.75%, 84.58%, and 83.92% in the ‘near,’ ‘far,’ and ‘farther’ conditions, respectively), a non-negligible portion of fixations occur beyond this range and cannot represent a continuation of the initial pre-stimulus fixation. Rather, they indicate that the observer’s gaze shifted away from the cue during the onset of the stimulus and before this fixation was logged, reflecting some preparatory or anticipatory behavior in response to the impending stimulus presentation.

It is interesting to note that 0th fixation durations show a clear trend based on the distance of the cue marker relative to the stimulus. In the ‘near’ condition, the mean 0th fixation duration was 612 ms (SD = 820 ms), long enough to suggest that observers started to engage with the visual content of the overlapping stimulus. In contrast, durations decrease rapidly as the stimulus moves farther from the marker and were found to be 443 ms (SD = 343 ms) in the ‘far’ condition and 393 ms (SD = 137 ms) in the ‘farther’ condition. At the very least, this pattern suggests that as the cue marker position becomes less visually coupled with the stimulus, observers move away from it to engage with the stimulus (i.e., the real visual content) more quickly.

### 3.2. Behavior of 1st Fixation

The middle row in Figure 5 shows the spatial distributions of the 1st fixation. Recall that by our numbering scheme, this is the fixation whose preceding saccade started after the onset of the stimulus. Two major separate clusters of fixations can be seen in the ‘far’ and ‘farther’ conditions. One cluster appears to be centered near the cue marker, whereas the other occurs closer to the stimulus center. In the ‘near’ condition, this bimodality is less noticeable. Even so, the consistent behavior in “shifting” from the 0th to the 1st fixation in the ‘far’ and ‘farther’ conditions suggests that a bimodality is also present in the ‘near’ condition and is less visible only because the two modes overlap.

A small proportion of 1st fixations (∼8%) remain within 1 degree VA from the cue marker across all conditions, indicating that, in most trials, the 1st fixation moves away from the marker, signaling stimulus exploration. Among these 8% of 1st fixations, a high proportion (∼70% in the ‘near’ condition, and 86–88% in the ‘far’ and ‘farther’ conditions) occur within 1 degree of their corresponding 0th fixation (Note that the latter may not necessarily be identical to the precise cue marker location). This pattern suggests that these 1st fixations reflect a continuation of the ongoing 0th fixation, representing corrective fixations or fixational microsaccades [26], rather than a newly programmed, stimulus-driven movement. The time-based analysis in Section 3.5 provides additional context for this interpretation and indicates that 1st fixations that remain close to the cue-marker position predominantly follow very short 0th fixation durations, whereas longer 0th fixation durations are associated with 1st fixations landing near the stimulus center.

Not unlike the 0th fixation, a pattern in fixation durations is observed for 1st fixations too, albeit much weaker. This time, mean durations were found to be 562 ms, 506 ms, and 481 ms, with standard deviations of 528 ms, 455 ms, and 452 ms in the ‘near,’ ‘far,’ and ‘farther’ conditions, respectively.

To determine whether the bimodality arises between or within observers, and thus resolving whether it emerges from inter-individual differences rather than a broad population phenomenon, we conducted a Linear Mixed Model (LMM) analysis on the 1st fixation distances separately for each marker-to-stimulus distance. In each LMM, the fixed intercept represents the overall average fixation distance across all trials and subjects within a condition. This is essentially the horizontal line fitted to all the data for that condition. In contrast, the random intercepts capture the subject-specific deviations from that overall average, indicating how much each observer’s mean fixation distance differs from the grand mean.

Table 1 summarizes the results of the LMM analysis for each condition, where, for instance, the fixed intercept of 10.2° in the Farther condition indicates that, on average, subjects fixate 2.96° from the stimulus center. The random intercept standard deviation of 1.01° represents how the average fixation distances of the observers vary around this general mean. However, the residual standard deviation of 3.36° is much larger, indicating that the variability within subjects (from trial to trial) is the dominant source of variation.

The relatively small random effect standard deviations across all conditions (ranging from 0.37° to 1.01°) compared to the larger residual standard deviations (ranging from 1.97° to 3.36°) suggest that while there are some individual differences, the subject-specific averages are fairly similar across observers. Most of the variability in fixation distances comes from trial-to-trial fluctuations rather than from a distinct subgroup of observers consistently fixating nearer the cue marker. Thus, the bimodality in 1st fixation behavior appears primarily as a trial-level phenomenon, rather than being driven by a subset of observers with markedly different behavior.

**Figure 6:**
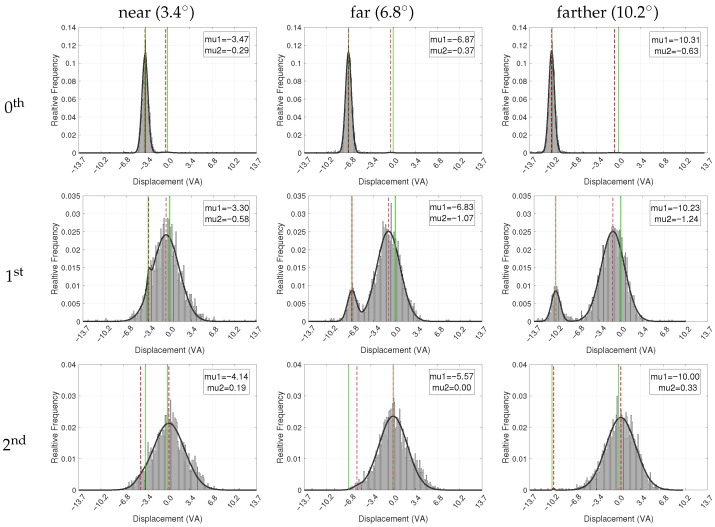
Histograms of horizontal fixation distances from the stimulus center, fitted with a two-component normal mixture distribution (black graph). The histograms are normalized, with the y-axis representing relative frequency. The 0th fixation refers to the gaze at the cue marker, setting the baseline for subsequent stimulus-directed fixations. Vertical green lines denote the stimulus center, represented by the origin and the marker location. Dashed red lines indicate the mean ($\mu_{1}$, $\mu_{2}$) of the fitted Gaussian components, corresponding to the cue-related and stimulus-centered fixation clusters, respectively. Distances to the left of the stimulus center are represented by negative values. The data presented consolidate both conditions where the cue marker is located to the left and to the right of the stimulus center. To achieve this, data from right-side cue marker conditions have been horizontally mirrored and combined with the left-side data.

Beyond this overall trial-level variability, a visual pattern in Figure 6, where the stimulus-centered component ($\mu_{2}$) consistently appears slightly left of the stimulus center, motivates examining the lateral structure of the 1st fixation distributions. Because these pooled distributions combine fixations from left-stimulus trials with mirrored fixations from right-stimulus trials, this apparent shift cannot be interpreted at face value. To clarify its origin, we repeated the mixture analysis separately for left and right conditions. This decomposition revealed a strongly symmetric pattern: when the stimulus appeared on the right, $\mu_{2}$ lies consistently to the left of the stimulus center, whereas when the stimulus appeared on the left, $\mu_{2}$ lies to its right. Thus, 1st fixations tend to undershoot the stimulus center in the direction of the incoming saccade, and the negative $\mu_{2}$ observed in the pooled data simply reflects such a direction-specific undershoot rather than a global leftward directional tendency. Consistent with this interpretation, the proportion of 1st fixations remaining at the cue location did not differ reliably between sides, and first-saccade magnitudes showed only small and inconsistent side differences across conditions.

### 3.3. Behavior of 2nd Fixation

Finally, the spatial distribution of the 2nd fixation is shown in the bottom row of Figure 5 and appears to have both higher spatial density near the center of the stimulus, and also a larger variance. In fact, qualitatively, the scatter plots suggest that by this fixation, the viewer has abandoned the cue marker to solely explore the stimulus. While the pattern is clear in the ‘far’ and ‘farther’ conditions, by extension it informs about the ‘near’ conditions too. In other words, it is highly likely that fixations within 1° VA from the cue position in the ‘near’ condition are part of the stimulus exploration rather than leftover effects of the cue marker.

### 3.4. Progression Toward the Center of Stimuli

Taken together, the fixation maps in Figure 5 reveal interesting population properties of fixations following the onset of the stimulus. In particular, observed scanpaths constitute either one or two fixations around the cue marker position before moving closer to the stimulus center. While time is not explicitly represented, the data illustrates that, on average, there is a gradual shift in fixation locations, moving closer to the stimulus center. Moreover, some scanpaths begin with only a short saccade that remains farther away from the stimulus center. This progression appears to complete by the 2nd fixation, with a unimodal distribution of fixations near the stimulus center. Importantly, this applied regardless of where the stimulus appeared on the screen.

To better understand the spatial dynamics of gaze behavior from the cue marker to the stimulus center, we focused on how the horizontal displacement between the fixations and the stimulus center varied across trial conditions. The horizontal displacement values from all trials were collected into histograms, followed by a normalization to obtain relative frequencies. Subsequently, these frequencies were fitted to a two-component normal mixture distribution (a.k.a. GMM) to obtain densities. Fitting was done using non-linear least squares, with the same initial conditions applied to all fixation numbers: one Gaussian component was initially centered at 0, representing the horizontal center of the stimulus, while the other component was initially centered 3.4°, 6.8°, and 10.2° VA to the left or right, respectively, representing the cue marker position. The initial weights of both components were set to 0.5 and all of their parameters (including their mean and variance) were made tunable during the optimization. Note that each weight indicates the proportion of fixations best accounted for by that component, with the two weights summing to 1, providing a descriptive summary of the visually evident bimodality in the data (rather than formal inferential analysis).

Figure 6 shows the displacement data and the fitted distribution for the different combinations of the fixation number and stimulus-to-marker distance. Similar to Figure 5, the presented data and models consolidate both conditions where the cue marker is located to the left and to the right of the stimulus center, after mirroring the right-side data and combining it with the left-side data.

The 0th fixation (top row) exhibits a single sharp dominant mode with an average mixture weight of 0.99 (where the other component is weighted insignificantly at two orders of magnitude smaller and essentially captures just noise). In the ‘near’, ‘far’, and ‘farther’ conditions, the mean value of the dominant component was 3.47°, 6.87°, and 10.31° VA to the left to the stimulus center, respectively, all well within the eye tracker measurement error.

The results for the 1st fixations reflect two modes, one around the cue marker and another around the stimulus center. Note how the bimodality shows up clearly in all cases, including the ‘near’ condition, although it was more difficult to discern in the corresponding fixation map in Figure 5. Quantitatively, the dominant component was less than 1° VA from the stimulus center in the ‘near’ condition and slightly more than this distance in the ‘far’ and ‘farther’ conditions, accounting for approximately 0.98, 0.89, and 0.89 of fixations, respectively. The other (cue marker) component was 3.30°, 6.83°, and VA away from the stimulus center, corresponding well to the actual marker-to-stimulus distance in each condition.

Finally, the modeling of the 2nd fixation (last row in the figure) essentially shows a single dominant component with an average weight of 0.99. Regardless of the distance between the marker and the stimulus center, this dominant component was centered at less than half a degree of VA from the center of the stimulus, indicating that, on average, the 2nd fixation indeed converged to the stimulus center, and certainly within the average tracking error measured during calibration.

Table 2 summarizes the overall horizontal fixation displacements from the stimulus center. As expected, mean values show a clear convergence toward the stimulus center across successive fixations. For the 1st fixation, the underlying distribution is bimodal (see Figure 6), and the intermediate mean reflects the mixture of cue-proximal and stimulus-centered fixations rather than a single mode.

### 3.5. Time-Based Analysis of Early Fixations

To further examine how early gaze behavior transitions from preparatory to stimulus-driven fixations, we analyzed fixation locations as a function of the duration of the preceding fixation, a type of analysis inspired by Rothkegel et al. [23]. Here, we related 0th fixation duration (recall from the Introduction that this is also the first saccade latency) to the landing position of the 1st fixation, and 1st fixation duration to the landing position of the 2nd. For this analysis, fixation durations were grouped into three bins: <150 ms, 150–350 ms, and >350 ms fixations. For each bin and each cue-to-stimulus distance condition (near, far, farther), we computed the mean distance of the subsequent fixation from the stimulus center.

**Figure 7:**
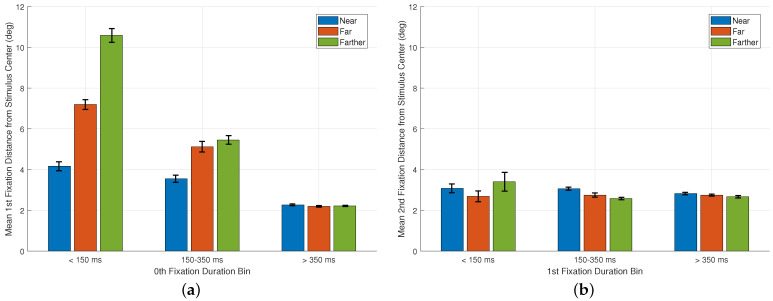
Time-based analysis of early fixation behavior. (**a**) Mean horizontal distance of the 1st fixation from the stimulus center as a function of the duration of the preceding 0th fixation. Duration values were grouped into three bins: <150 ms, 150–350 ms, and >350 ms. Separate bars are shown for the near, far, and farther cue-to-stimulus distance conditions. (**b**) Mean horizontal distance of the 2nd fixation from the stimulus center as a function of the duration of the preceding 1st fixation, using the same duration bins and condition labels. Error bars represent ±1 SE.

Figure 7a shows the mean distance of the 1st fixation from the stimulus center as a function of the duration of the 0th fixation. Across all conditions, durations < 150 ms are followed by 1st fixations that remain well away from the stimulus center with mean distances of 4.17°, 7.19°, and 10.58° for the near, far, and farther conditions, respectively. In contrast, durations above 150 ms, and especially those exceeding 350 ms, are associated with 1st fixations that land closer to the stimulus center, with mean distances converging to approximately ∼2–3° across all conditions once latency exceeds 350 ms. This pattern supports the interpretation that very short-latency eye movements, typically initiated <150 ms after stimulus onset, reflect early movements that do not yet incorporate substantial stimulus information.

Figure 7b presents a similar analysis for the 2nd fixation. Here, the mean distance of the 2nd fixation from the stimulus center is shown as a function of the duration of the 1st fixation. Whereas Rothkegel et al. [23] showed that longer 1st fixations reduce the likelihood of the next fixation landing near the image center, our data shows that 2nd fixations tend to land close to the stimulus center across all 1st fixation durations, with only minor variations across bins and conditions. This difference can be understood in light of the methodological context: in our paradigm, the 1st fixation frequently undershoots the stimulus center due to the large cue-to-stimulus distance, making the 2nd fixation function as a corrective movement whose landing position is driven primarily by spatial error rather than by the duration of the preceding fixation. These differences likely reflect the distinct viewing geometry and timing in the two paradigms, highlighting how the initial steps of scene exploration can depend strongly on the specific experimental context and motivating the use of time-resolved analyses when interpreting early gaze behavior.

## 4. Discussion

One of the goals of the present study was to determine whether the eye is drawn to the center of a stimulus regardless of its offset from the display center, including conditions where no part of the stimulus overlaps the latter point. This differs from an earlier approach [24] where such overlaps confounded the possible conclusions. Additionally, the prior art placed the cue markers in the corners of the display, thus maintaining a fixed distance from its center. That methodological choice prohibited exploring the effect of that distance on the center bias, and at the same time introduced constraints on post-cue fixations (as fixating away from the corners outside the display was unlikely). Our study avoids the latter while also making the offset between the display center and the cue marker a truly independent variable, thus facilitating an investigation of how the distance between the cue and the stimulus affects the center bias.

**Figure 8:**
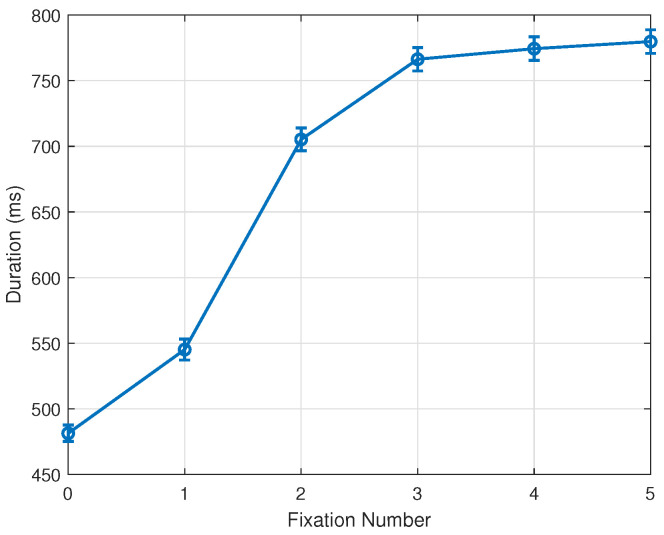
Mean and standard error of fixation durations. 0th and 1st fixations, often outside the stimulus boundary, show markedly shorter durations. Bars represent 1 SE.

Following these changes in methodology, our experimental results indicate a clear fixation bias and tendency toward the stimulus center. As reported, this pattern emerges by the 1st fixation and completes in the 2nd fixation after the onset of the stimulus. Further analysis of fixation durations provides additional insights into this phenomenon. While Section 3.1 and Section 3.2 discuss fixation durations by stimulus distance condition, Figure 8 pools all data of each fixation across all conditions. Clearly, the 0th and 1st fixations are in general markedly shorter than later ones. A plausible explanation for this observation is that many early fixations, particularly in the ‘far’ and ‘farther’ conditions, fall outside the stimulus region, where there is little reason to fixate for long. At the same time, such an overall increase in fixation duration across the early phase of scene viewing has already been documented in the literature (e.g., Nuthmann [27]). Our results therefore replicate this well-established temporal trend, while demonstrating it under conditions in which the earliest fixations may occur outside the stimulus due to the offset configuration of our paradigm.

This corresponds well with the observation that many early fixations, especially in the ‘far’ and ‘farther’ conditions, are located outside the stimulus region, where there is little reason to fixate for long. We note that the grand average of fixation duration across all fixations, conditions, trials, and subjects indicates approximately 1.7 fixations per second over the 3-s trial period. Consistent with these observations, the time-based analysis in Section 3.5 further shows that short-latency eye movements tend to preserve cue-related behavior, whereas longer fixation durations prior to each saccade reliably yield the center-directed pattern characteristic of stimulus-driven exploration.

Our main finding is consistent with previous research indicating that the abrupt appearance of a visual stimulus attracts visual attention regardless of the visual content of the stimulus [28,29]. Additionally, the results indicate (to our knowledge, for the first time) that the distance between the marker and stimulus modulates the initial expression of the center bias, in that larger required saccade amplitudes lead to greater undershooting and dispersion in the 1st fixation. These findings also support the conclusions by Bindemann [24] about the effect of the stimulus location on the tendency toward the center. However, while that work attributed the difficulty of reaching the center of the stimulus to a tendency toward the center of the screen, our analysis reveals that this difficulty varies as a function of cue marker distance from the center of the stimulus. As a result, the difficulty in reaching the stimulus center observed in the prior art could also be related to cue position. In other words, the difficulty in reaching the stimulus center can also reflect the influence of cue position, rather than being driven solely by the display center. Importantly, although our results highlight the strong influence of the stimulus center, they do not rule out a potential contribution of the display center. The display center remains a possible contributing factor, and clarifying the interplay between stimulus center and display center biases remains an important direction for future research, especially under different experimental viewing conditions.

It is noteworthy that in Bindemann [24], the tendency to fixate on the center of the stimulus is manifested only in the first fixation. In contrast, our results show increased fixation density toward the center of the stimulus beginning in the 1st fixation and continuing through the 2nd. This difference could be attributed, at least in part, to the particular way that the raw tracking data were processed into fixations and saccades (see the Section 1). More specifically, Bindemann pre-processed eye movements by merging fixations shorter than 80 milliseconds with the fixation immediately preceding or following, provided they were within 0.5° VA. They further refined the data by omitting brief fixations that fell outside this half-degree range. The fixations in our experiment, on the other hand, were determined by the eye-tracker software based on the velocity of saccades and without any additional post-processing.

Clearly, what constitutes a fixation is a fundamental factor in most eye-movement-related research and we encourage researchers to continue investigating how to better parse the raw tracking data to fixations and saccades and how the parsing approach affects the obtained insights. This could be facilitated, for example, by analyzing not only the fixations and saccades but also the raw gaze samples recorded by the eye-tracker before they are processed into a scan path. To facilitate such future research, these raw data from our own study are provided to any interested researcher in a publicly available repository [30].

## Figures and Tables

**Table 1:** Summary of LMM parameters for 1st fixation by marker-to-stimulus distance.

Condition	Fixed Intercept	Random Effect SD	Residual SD
near (3.4°)	1.91	0.37	1.97
far (6.8°)	2.42	0.81	2.63
farther (10.2°)	2.96	1.01	3.36

**Table 2:** Mean ± SD horizontal displacement (° VA) from the stimulus center by fixation number and marker displacement condition. Negative values indicate fixations toward the cue marker. For the 1st fixation, means reflect the mixture of cue- and stimulus-centered fixations due to bimodality.

	Near (3.4°)	Far (6.8°)	Farther (10.2°)
0th	−3.36±2.20	−6.76±1.75	−10.28±1.67
1st	−0.52±2.72	−1.65±3.27	−2.51±3.84
2nd	0.21±2.86	0.22±2.88	0.21±2.57

## Data Availability

All raw data from our study are provided to any interested researcher in a publicly available repository [30].
